# DNA barcoding reveals seasonal shifts in diet and consumption of deep-sea fishes in wedge-tailed shearwaters

**DOI:** 10.1371/journal.pone.0195385

**Published:** 2018-04-09

**Authors:** Taketo Komura, Haruko Ando, Kazuo Horikoshi, Hajime Suzuki, Yuji Isagi

**Affiliations:** 1 Graduate School of Agriculture, Kyoto University, Sakyo-ku, Kyoto, Japan; 2 Center for Environmental Biology and Ecosystem Studies, National Institute for Environmental Studies, Onogawa, Tsukuba, Ibaraki, Japan; 3 Institute of Boninology, Nishi-machi, Chichijima, Ogasawara, Tokyo, Japan; University of Hyogo, JAPAN

## Abstract

The foraging ecology of pelagic seabirds is difficult to characterize because of their large foraging areas. In the face of this difficulty, DNA metabarcoding may be a useful approach to analyze diet compositions and foraging behaviors. Using this approach, we investigated the diet composition and its seasonal variation of a common seabird species on the Ogasawara Islands, Japan: the wedge-tailed shearwater *Ardenna pacifica*. We collected fecal samples during the prebreeding (*N* = 73) and rearing (*N* = 96) periods. The diet composition of wedge-tailed shearwater was analyzed by Ion Torrent sequencing using two universal polymerase chain reaction primers for the 12S and 16S mitochondrial DNA regions that targeted vertebrates and mollusks, respectively. The results of a BLAST search of obtained sequences detected 31 and 1 vertebrate and mollusk taxa, respectively. The results of the diet composition analysis showed that wedge-tailed shearwaters frequently consumed deep-sea fishes throughout the sampling season, indicating the importance of these fishes as a stable food resource. However, there was a marked seasonal shift in diet, which may reflect seasonal changes in food resource availability and wedge-tailed shearwater foraging behavior. The collected data regarding the shearwater diet may be useful for *in situ* conservation efforts. Future research that combines DNA metabarcoding with other tools, such as data logging, may provide further insight into the foraging ecology of pelagic seabirds.

## Introduction

Seabirds are critical organisms within marine ecosystems as they are top-order predators in hierarchical food webs. Thus, seabird populations have often been used as marine ecosystem indicators [1–4]. The diets of pelagic seabirds, which may reflect fluctuations in available marine food resources over their broad foraging ranges, are also useful indicators of marine biological productivity [5,6]. Most studies of seabird diets have been conducted during only the rearing period, as conventional methods are unsuitable for assessing their diets during other periods, such as the prebreeding season [7]; information on seabird diets during the prebreeding season remains insufficient because conventional methods are unsuitable under these conditions. To use seabird diets as an indicator to monitor seasonal shifts in marine biological productivity, it is necessary to conduct diet analyses of seabirds on a wider temporal scale, including the prebreeding and breeding seasons. DNA metabarcoding of fecal samples is a noninvasive and sensitive method for identifying prey taxa [8,9]. This method does not place stress on the animals to determine their stomach contents; the metabarcoding method also requires less effort than conventional morphological analyses [7]. With the development of high-throughput sequencing techniques, it is now possible to recover DNA sequences from fecal samples relatively easily [10,11]. In recent years, this method has been mainly used in dietary studies of large terrestrial mammals [12]. Continuous dietary research that applies noninvasive and sensitive DNA metabarcoding to seabirds is likely to be useful for monitoring marine ecosystems with very little impact on birds. However, only a few such studies have targeted marine animals, such as penguins and seals [13,14], and only a limited number of Procellariidae species have been examined using DNA metabarcoding [15–18].

The wedge-tailed shearwater *Ardenna pacifica* is a potential indicator of Pacific marine ecosystems due to its wide distribution in the subtropical Pacific and Indian Oceans and its large population size [19]. In this study, we focused on the population on the Ogasawara Islands, which constitute a subtropical oceanic island chain located 1,000 km south of the main island of Japan, and these islands are the only wedge-tailed shearwater breeding colony site in Japan [20]. The productivity of the subtropical ocean surface is lower than that of northern oceans [21], and it would be valuable to determine if shearwaters consume only surface nekton or if they also consume deep-sea animals and how they change their diet as the food resources change. Nekton communities generally exhibit seasonal migration and fluctuation [22–24], which may affect the feeding behavior of seabirds. Indeed, other Procellariidae species exhibit seasonal shifts in foraging behavior [25,26], and provision behaviors of seabirds have been shown to change during the rearing period [27]. Therefore, it is important to compare the diets of wedge-tailed shearwaters between two breeding phases to investigate such shifts.

Using a DNA metabarcoding approach, we investigated the dietary composition of the wedge-tailed shearwater during the prebreeding and rearing periods to compensate for the deficiency of the current knowledge of shearwater diet during the prebreeding period and its seasonal dietary shifts. In addition, we surveyed the diets of wedge-tailed shearwaters to estimate the foraging behavior of this species in relation to food resource availability and the possible contribution of the metabarcoding approach to marine ecosystem monitoring.

## Materials & methods

### Ethics statement

Fecal samples were collected on the ground at a wedge-tailed shearwater breeding site in a national forest on Minamijima Island, Japan under an entry license from the Forest Agency of Japan. During sample collection, we did not capture any birds and avoided damaging their nests.

### Study site

Our study site was located on the Ogasawara Islands, which were listed as a World National Heritage Site in 2011. This island chain is a breeding site for 16 species of seabirds, including five Procellariidae species [28,29]. Of these species, 10 are included on the Red List of the International Union for Conservation of Nature and/or the Ministry of the Environment of Japan [30,31]. This study was conducted on Minamijima Island (27°02’N, 142°10’E), which contains one of the largest colonies of wedge-tailed shearwaters on the Ogasawara Islands.

### Target species

The wedge-tailed shearwater *Ardenna pacifica* is a medium-sized species of the Procellariidae family that breeds on oceanic islands in the tropical Pacific and Indian Oceans as well as on the east and west coasts of Australia. The global wedge-tailed shearwater population is estimated to be more than 5,200,000 individuals [19], and the number of breeding pairs in Japan is estimated to be 10,000–100,000 [32]. Although the population of this species is large, there are concerns that its numbers are declining owing to unsustainable levels of exploitation, persecution, predation by invasive species, and overfishing [19].

Although the wedge-tailed shearwater is a medium-sized species, it is the largest species of Procellariidae on the Ogasawara Islands. In these islands, wedge-tailed shearwaters lay eggs in June or July, incubate the eggs for approximately 50 days, and then rear the chicks for approximately 50 days (Kazuo Horikoshi and Hajime Suzuki, personal observation).

### Fecal sampling and DNA extraction

We collected 73 and 96 fecal samples in the early mornings when the adults were absent in May 2015 (prebreeding period) and September 2015 (rearing period), respectively, on rocky limestone and grass in an exclusive wedge-tailed shearwater colony. We took care not to mix uric acid into fecal samples during collection. These sampling criteria have been deemed valid for the efficient recovery of prey DNA [18,33]. Most of the fecal samples were dried naturally in the field. The collected feces were stored at –30°C until DNA extraction. DNA from samples >5 mg and <20 mg in fecal dry weight was extracted using the QIAamp DNA Stool Mini Kit (QIAGEN, Venlo, the Netherlands).

### DNA barcoding

Following previous studies based on stomach analysis conducted in Hawaii [34], we assumed that the main prey of wedge-tailed shearwaters were fishes and mollusks. Universal primer pairs were used to amplify the mtDNA of the 12S and 16S regions of fishes and mollusks, respectively (Table 1). These regions have frequently been used for the amplification and identification of DNA of vertebrates and cephalopods in diet studies [8,35,36]. The forward primers were tagged with a multiplex identifier (MID) [37] to track the sequences resulting from each sample. We designed a blocking primer specific to Procellariidae sequences by modifying the primers used in a previous study [38]. Polymerase chain reaction (PCR) amplification was conducted using a Qiagen Multiplex PCR kit (Qiagen), and each 10 μL sample of the total reaction mixture volume targeting the 12S region (fishes) contained 1 μL of extracted DNA, 6 μL of Multiplex PCR Master Mix, 0.24 μmol/L of each primer pair, and 1.2 μmol/L of blocking primer. The PCR conditions were denaturation for 15 min at 95°C, 35 cycles of 30 s at 94°C, 1.5 min at 57°C, and 1 min at 72°C, with a final cycle of 10 min at 72°C. Each 10 μL sample of the total reaction mixture volume targeting the 16S region (mollusks) contained 1 μL of extracted DNA, 6 μL of Multiplex PCR Master Mix, and 0.24 μmol/L of each primer pair. The PCR conditions were denaturation for 15 min at 95°C, 40 cycles of 30 s at 94°C, 1.5 min at 47°C, and 1 min at 72°C, with a final cycle of 10 min at 72°C. The PCR products were purified using exonuclease I and shrimp alkaline phosphatase (exo/SAP; Takara, Shiga, Japan and Promega, Madison, WI), an E-Gel Agarose Gel Electrophoresis System (Thermo Fisher Scientific, Waltham, MA, USA) and Agencourt AMPure XP (Beckman Coulter). The presence of a PCR product of suitable length was confirmed using the MultiNA Microchip Electrophoresis System (Shimadzu). Sequencing was performed using an Ion Torrent Personal Genome Machine (PGM) system with the Ion PGM 200 Sequencing Kit and the Ion 318 Chip (Thermo Fisher Scientific, Waltham, MA, USA) following the manufacturer’s instructions. We used Claident software [39] to separate the obtained sequences into samples using the MID tags and filter the sequences. Filtering excluded sequences that met at least one of the following DNA barcoding conditions: 1) sequence length <100 bp, 2) mean Phred-like quality value <20, and 3) minimum MID tag quality value <20. We then trimmed the 30 lowest quality tails, leaving three continuous sequences with a minimum quality value of 20. DNA barcoding was performed for each filtered sequence through a global BLAST search using BLAST2Go [40]. We identified a prey fish and a prey mollusk by referring to the National Center for Biotechnology Information (NCBI) sequence database. The correspondence of each sequence to the reference sequences was estimated based on the BLASTN algorithm with an E-value threshold of <10^−25^. Almost none of the 16S sequences for mollusk detection could be assigned using this threshold; therefore, the match of each 16S sequence to the reference sequences was estimated using an E-value threshold of <10^−5^. We accepted genera of the species assigned the lowest E-value and highest similarity score [41]. We identified samples to the genus level, even if some sequences matched at the species level, because the current sequence database does not include all prey species known to be distributed in the wedge-tailed shearwater foraging range and because knowledge of these prey species is incomplete. Therefore, species-level identification of prey species at our study site might be unreliable, and we cannot determine whether species that are top hits in BLAST are prey or related species that share 12S or 16S sequences. The development of a comprehensive species list and sequence database for the waters surrounding the Ogasawara Islands might enable future studies to estimate prey to the species level.

**Table 1:** Sequences of the primer pairs used in this study. The lengths of the amplified fragments (excluding primers) with 12SV5 and Mol-16S were 105–121 and 102–143 bp, respectively.

Primer name	Region	Sequence (5'-3')	No. of prey taxa identified	Reference
F_12SV5	mtDNA 12S	TAGAACAGGCTCCTCTAG	31	[43]
R_12SV5	mtDNA 12S	TTAGATACCCCACTATGC		[43]
Mol-16S-F1	mtDNA 16S	MCTTWTAAWTKRAGGCTAGA	9	[44]
Mol-16S-R2	mtDNA 16S	MYYAGGGTCTTSTTGTC		[44]
blocking primer for shearwaters	mtDNA 12S	CTATGCTTAGCCCTAAATCTTGATACTTACC-C3		

If two or more taxa were assigned the same score for a given sequence, the sequence was assigned to the lowest taxonomic level that included both taxa. Following identification of prey taxa, we categorized each prey taxon into its habitat group, i.e., epipelagic, mesopelagic, benthic, bathypelagic, or reef, using “Fishes of Japan with pictorial key to the species” [42] as a reference.

### Statistical analysis

To avoid misidentification of prey due to sequencing errors and contamination, we selected a threshold for removing prey with low read frequencies (<2% in each sample) based on the results of preliminary experiments on food-controlled fecal samples. This threshold was within a valid range established in previous studies [18,45]. Rarefaction curves were used to confirm the sufficiency of obtained sequences and sampling effort using the vegan package [46] in the R ver.3.2.2 software [47]. Read-based rarefaction curves were created by performing repeated random resampling of the pool of N reads and then plotting the average number of operational taxonomic units (OTUs) detected in each group of reads. According to the read-based rarefaction curves, 10 reads were predicted to cover most of the potential OTUs. We retained samples containing more than nine reads. To eliminate the effects of variation in the reads among samples, we equalized the number of reads per sample to 10. Next, we created sample-based rarefaction curves for each period. After that, we calculated the relative read abundance of the sequences (RRA) and the frequency of occurrence (FOO). Although the proportion of sequence reads may not have correctly reflected the mass of consumed prey due to differences in prey DNA density and survival of DNA during digestion [48], it was reported in previous diet studies that the sequences of prey taxa that were fed in a large amounts were frequently detected [49–51]. In addition, some studies have suggested that the proportion of sequences provides semi-quantitative data [52,53]. The frequency of sequence reads is also used to reduce the probability of misidentification by recognizing secondary ingestion, i.e., food items consumed by prey species [54]. We calculated the sequence rates of each food taxon in each sample, and then performed a hierarchical cluster analysis of the sequence rates of fishes and mollusks using Ward’s method. To visualize diet composition distances among fecal samples, we used nonmetric multidimensional scaling (NMDS) in the vegan package.

## Results

### Identification of fishes and mollusks in feces using the 12S and 16S regions

We obtained 125,040 reads (105–121 bp) from the 12S region targeting fishes and 28,235 reads (102–143 bp) from the 16S region targeting mollusks. After excluding sequence errors and sequences identified as non-prey items (birds, non-oceanic species, and human beings), 86,666 reads of the 12S region (fish) and 28,218 reads of the 16S region (mollusks) remained. There were 26 samples from the prebreeding period with more than nine reads of 12S sequences, 18 samples from the prebreeding period with more than nine reads of 16S sequences, 89 samples from the rearing period with more than nine reads of 12S sequences, and 44 samples from the rearing period with more than nine reads of 16S sequences. According to the rarefaction curves, most samples converged (S1 Fig), and the number of samples may have been sufficient (S2 Fig). In the 12S region, the assigned OTUs were taxonomically separated into 25 genera, four families, one order, and one superorder of prey fishes (Table 2). In the 16S region, the assigned OTUs were taxonomically separated into three genera, three families, two orders, and one superorder of prey mollusks (Table 3).

**Table 2:** List of vertebrate taxa identified based on 12S sequences and the corresponding number of reads.

Food items	N reads	N reads after equalization	RRA (%)	FOO (%)	Habitat
**Myctophidae**					
*Benthosema*	35060	327	28.43	34.92	mesopelagic
*Diaphus*	210	1	0.09	0.79	mesopelagic
*Nannobrachium*	6	0	0	0	mesopelagic
*Ceratoscopelus*	1	0	0	0	mesopelagic
**Exocoetidae**					
*Cheilopogon*	13651	264	22.96	39.68	epipelagic
*Exocoetus*	2110	35	3.04	3.97	epipelagic
*Cypselurus*	29	0	0	0	epipelagic
**Mullidae**					
*Mulloidichthys*	11502	183	15.91	24.6	benthic and reef
*Parupeneus*	546	9	0.78	3.97	benthic and reef
Unidentified	39	12	1.04	1.59	benthic and reef
**Scopelarchidae**					
*Scopelarchoides*	7109	86	7.48	12.7	mesopelagic
**Scombridae**					
*Thunnus*	6925	72	6.26	11.11	epipelagic
**Molidae**					
*Ranzania*	2435	53	4.61	6.35	epipelagic
**Gempylidae**					
*Gempylus*	2110	13	1.13	3.17	mesopelagic and epipelagic
*Diplospinus*	68	0	0	0	mesopelagic
**Clupeidae**					
*Sardinops*	1532	8	0.70	0.79	epipelagic
**Serranidae**					
*Epinephelus*	1179	1	0.09	0.79	reef
**Carangidae**					
Unidentified	617	2	0.17	0.79	epipelagic
**Coryphaenidae**					
*Coryphaena*	426	16	1.39	2.38	epipelagic
**Chaetodontidae**					
Unidentified	394	10	0.87	1.59	reef
**Nomeidae**					
*Psenes*	317	5	0.43	1.59	epipelagic and benthic
**Engraulidae**					
*Encrasicholina*	278	10	0.87	1.59	epipelagic
**Gonostomatidae**					
*Diplophos*	160	0	0	0	mesopelagic
**Balistidae**					
Unidentified	124	3	0.26	0.79	reef
**Synodontidae**					
*Synodus*	72	19	1.65	1.59	benthic
**Rhynchactis**					
*Rhynchactis*	36	2	0.17	0.79	mesopelagic
**Kyphosidae**					
*Kyphosus*	34	11	0.96	1.59	epipelagic and reef
**Melamphaidae**					
*Scopeloberyx*	25	2	0.17	0.79	bathypelagic and mesopelagic
**Opisthoproctidae**					
*Opisthoproctus*	7	5	0.43	0.79	bathypelagic and mesopelagic
**Unidentified Acanthopterygii**	14	1	0.09	0.79	
**Unidentified Perciformes **	13	0	0	0	
Taxa of host species & contaminations	N reads				
*Puffinus*	34805				
*Homo sapiens*	5034				
*Nipponia*	47				
*Sula*	46				
*Pterodroma*	36				
Siniperca	18				
Cobitidae	9				
*Bos*	7				
*Rattus*	4				
*Pluvialis*	3				

**Table 3:** List of mollusk taxa identified based on 16S sequences and the corresponding number of reads.

Food items	N reads	N reads after equalization	RRA (%)	FOO (%)	habitat
**Decapodiformes**					
Unidentified	392	9	1.45	1.59	
**Oegopsida**					
Unidentified	191	3	0.48	1.59	
**Ommastrephidae**					
*Ommastrephes*	21419	165	26.61	14.29	mesopelagic and epipelagic
*Sthenoteuthis*	5139	420	67.74	34.92	mesopelagic and epipelagic
*Nototodarus*	240	10	1.61	0.79	mesopelagic and epipelagic
**Opheliidae**					
Unidentified	518	10	1.61	1.59	benthic
**Octopoda**					
Unidentified	9	0	0	0	benthic
**Octopodidae**					
Unidentified	269	2	0.32	0.79	benthic
**Conidae**					
*Conus*	41	1	0.16	0.79	benthic
Taxa of contaminations	N reads				
Photobacterium	12				

### Diet comparisons between the prebreeding and rearing periods

**Fig 1:**
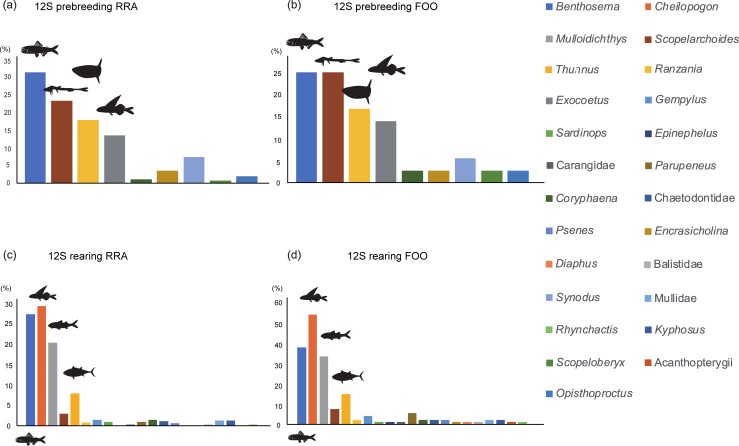
Comparison of fish prey composition between breeding phases. RRA: number of sequence reads of a particular food taxon divided by the number of total sequence reads of the samples used in statistical analysis, FOO: number of samples that included a particular food taxon divided by the total number of samples used in the statistical analysis. (a) Prebreeding period (RRA), (b) prebreeding period (FOO), (c) rearing period (RRA), and (d) rearing period (FOO).

**Fig 2:**
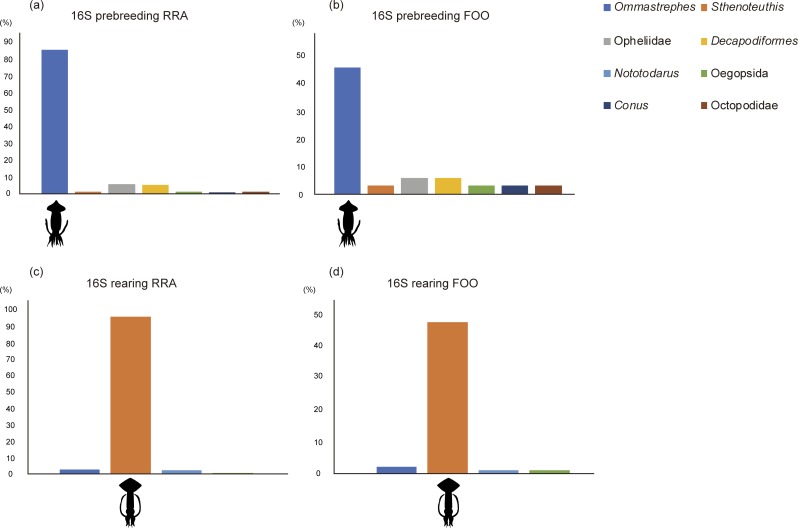
Comparison of mollusk prey composition between breeding phases. (a) Prebreeding period (RRA), (b) prebreeding period (FOO), (c) rearing period (RRA), and (d) rearing period (FOO).

During the prebreeding period, *Benthosema* (RRA = 31.15%, FOO = 25.71%), *Scopelarchoides* (RRA = 23.08%, FOO = 25.71%), *Ranzania* (RRA = 17.69%, FOO = 17.14%), and *Exocoetus* (RRA = 13.49%, FOO = 14.29%) were the major fish prey items, accounting for approximately 85% of all sequences (Fig 1A and 1B). *Scopelarchoides* and *Benthosema* are deep-sea fishes [42], and these results therefore indicate frequent consumption of deep-sea fishes by wedge-tailed shearwaters. *Ommastrephes* accounted for nearly all mollusk prey (RRA = 85.56%, FOO = 45.71%) (Fig 2A and 2B). During the rearing period, diet composition was distinct from that during the prebreeding period. *Cheilopogon* (RRA = 29.66%, FOO = 54.95%), *Benthosema* (RRA = 27.64%, FOO = 38.46%), *Mulloidichthys* (RRA = 20.56%, FOO = 34. 07%), and *Thunnus* (RRA = 8.09%, FOO = 15.38%) comprised the main fish prey items, accounting for approximately 86% of all sequences (Fig 1C and 1D). *Sthenoteuthis* accounted for the most mollusk prey species (RRA = 95.00%, FOO = 47.25%) (Fig 2C and 2D).

### Variation in prey composition among fecal samples

**Fig 3:**
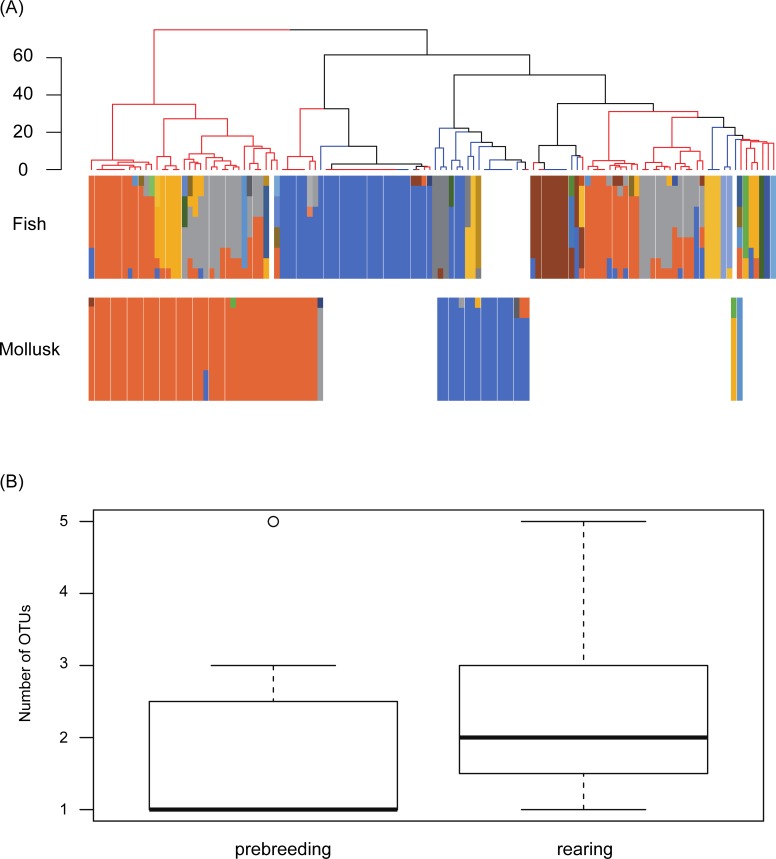
Diet dissimilarity between prebreeding and rearing. (A) Individual diet samples displayed as a dendrogram obtained from a cluster analysis of prey item sequences. The blue and orange tips in the dendrogram indicate fecal samples collected during the prebreeding and rearing periods, respectively. The column graphs below the dendrogram indicate the fish and mollusk compositions of the sample. The color of each taxon is as shown in Figs 1 and 2. (B) Box plot comparison of the number of fish operational taxonomic units (OTUs) per fecal sample between breeding phases. We identified samples to the genus level even if some sequences matched at the species level because the current sequence database does not cover all prey species distributed in the wedge-tailed shearwater foraging range. The left plot depicts the samples collected during the prebreeding period, and the right plot depicts the samples collected during the rearing period. The bold lines in the box indicate the median, and the edges of the box indicate the quartiles. A circle in the prebreeding plot indicates an outlier.

Inter-feces variability of the prey composition was assessed using cluster analysis, and all fecal samples were roughly divided into two clusters (Fig 3A). The observed variability may have been caused by prey composition differences among samples. The average numbers of taxa per sample from the prebreeding and rearing periods were 1.77 ± 0.99 (standard deviation [SD]) and 2.34 ± 1.09 (SD) (Fig 3B), respectively. There was a significant difference in the average number of taxa per sample between the periods (*t*-test, *P* < 0.05).

**Fig 4:**
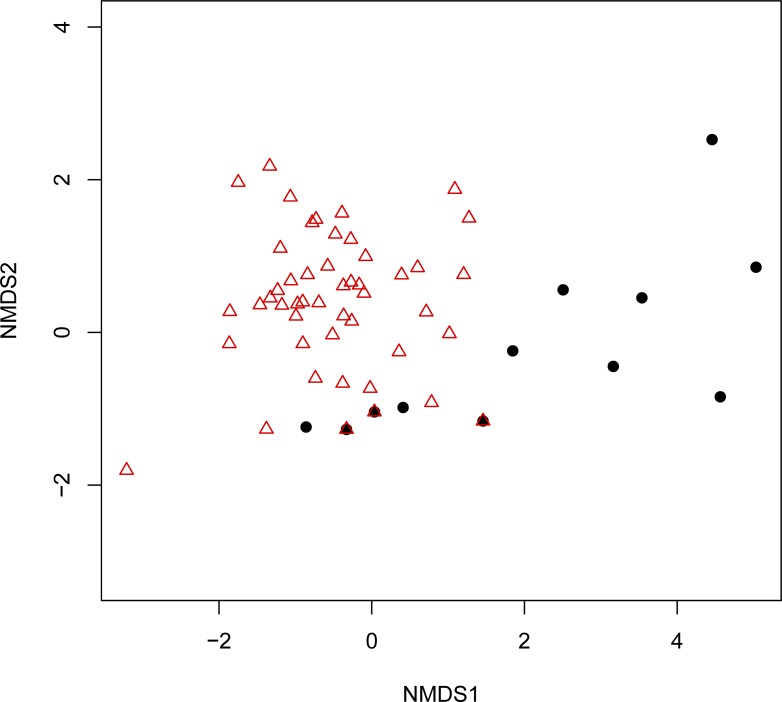
Differences in diet among fecal samples determined using nonmetric multidimensional scaling (NMDS). NMDS was based on the Bray–Curtis dissimilarity in the read rates of individual samples. Samples were classified by breeding stage. The black circles indicate prebreeding samples and the red triangles indicate rearing samples.

The NMDS analysis revealed differences in diets between the samples from the prebreeding and rearing periods (permutational multivariate analysis of variance [perMANOVA] test, *P* < 0.01; beta dispersion test, *P* = 0.1947) (Fig 4). Prebreeding samples tended to be distributed on the right side of the distribution, and rearing samples were distributed on the left side (Fig 4).

## Discussion

### Deep-sea fishes in wedge-tailed shearwater fecal samples

The number of samples and reads obtained from the 12S and 16S sequences seemed to be sufficient to assess diet composition, according to the rarefaction curves (S1 and S2 Figs).

The results of the present study indicate frequent deep-sea fish consumption by wedge-tailed shearwaters. The dominant fish taxa detected in the samples, such as *Benthosema* and *Scopelarchoides*, were mesopelagic nekton [42]. The detected result partly corresponds to a morphological study targeting wedge-tailed shearwaters conducted in Hawaii [34]; however, mesopelagic fishes were more frequently detected in the present study than in the previous study. This difference may be due to the high resolution of the DNA metabarcoding approach and/or the differences in foraging behavior of wedge-tailed shearwaters related to marine productivity or abundance of underwater predators. Due to the methodology differences, it is difficult to identify the actual reason for the differences in the estimated diets of wedge-tailed shearwaters in Hawaii and the Ogasawara Islands. Metabarcoding diet analyses in both breeding sites may reveal the detailed differences in foraging habits. There are four plausible explanations for the importance of deep-sea fishes in the sea around the Ogasawara Islands in shearwater diets.

First, shearwaters may consume the spawn or larvae of deep-sea fishes, which float at the sea surface. In this case, seasonal changes in the consumption of *Benthosema* and *Scopelarchoides* may be attributed to changes in the abundance of their spawn or larvae throughout their life cycles. Second, shearwaters may feed at night when *Benthosema* and *Scopelarchoides* swim upward to the sea surface (i.e., diel vertical migration) [42]. Previous studies investigated the foraging behavior of Procellariiformes using data loggers, geolocators, and global positioning systems (GPS). For example, it was reported that shy albatrosses (*Diomedea cauta*) and streaked shearwaters (*Calonectris leucomelas*) land on the water during both day and night but dive more frequently in the daytime than during the nighttime [55,56]. On the other hand, it has been reported that a Procellariidae species in the Azores, Bulwer’s petrel (*Bulweria bulwerii*), consumes mesopelagic nekton at night [57], and there is an instance of nocturnal foraging behavior by wedge-tailed shearwaters [58]; it is likely that wedge-tailed shearwaters on the Ogasawara Islands also forage at night. In a previous study that investigated the nekton biomass in the seas surrounding the Ogasawara Islands using ultrasonic fish finders, it was reported that nekton density was lower during the daytime than at night [59], suggesting that wedge-tailed shearwaters may forage at night because prey are more abundant. Third, shearwaters may consume deep-sea fishes that escape from underwater predators to the surface. It is known that the feeding behavior of wedge-tailed shearwaters is related to the distribution of subsurface predators such as yellowfin and skipjack tunas [60]. Underwater predators such as yellowfin tuna, skipjack tuna, dolphin fish and swordfish are distributed in the Ogasawara waters [42], and mesopelagic nekton was detected in the gut contents of yellowfin tunas and swordfishes [61,62]. Therefore, mesopelagic fishes that escape from underwater predators to the surface may be available for wedge-tailed shearwaters.

It is also possible that shearwaters consumed fishes discarded from fishing boats. It has been reported that some shearwater species change their foraging behavior in the presence of fishery discards [63–67]. However, trawl net fishing, in which fish with low commercial value are discarded, is not performed in the seas around the Ogasawara Islands because the shelf area is too small. It is therefore unlikely that wedge-tailed shearwaters depend on discarded fishes.

*Benthosema* and *Scopelarchoides* were detected in high frequencies throughout the sampling season, and the prey items held the first and second highest ranks during the prebreeding period, and the second and fifth ranks during the rearing period according to both RRA and FOO indicators (Fig 1). This result indicates that deep-sea fishes are stable and important food resources for wedge-tailed shearwaters.

### Squids found in wedge-tailed shearwater feces

The taxa that accounted for the largest proportion of mollusk prey, *Ommastrephes* and *Sthenoteuthis*, both belong to the family Ommastrephidae. A previous study collected large numbers of families such as Enoploteuthidae and Cranchiidae by trawl nets in seas adjacent to the Ogasawara Islands [59]. However, these families were not detected in the present study. To confirm whether these families can be amplified by the universal primer used in this study, we analyzed the 16S sequence data of 10 species each of Enoploteuthidae and Cranchiidae selected from the NCBI database. In all species, except for one from each family, all sequences included the universal primer sequence. This result suggests that wedge-tailed shearwaters selectively consumed Ommastrephidae. In actuality, this result corresponds to a previous study that targeted wedge-tailed shearwaters in Hawaii [34]. However, no comprehensive sequence database includes all local mollusk species from the Ogasawara Islands. Thus, we could not determine whether some Enoploteuthidae and Cranchiidae species went undetected due to primer mismatches. Further development of the nucleotide database may improve the accuracy of these results.

### Diet comparisons between the prebreeding and rearing periods

Several studies using geo-locators or GPS have revealed seasonal shifts in foraging behavior in marine birds [68–71]. For example, the durations of foraging excursions of Manx shearwaters (*Puffinus puffinus*) varied greatly with breeding stage [69], and the foraging trip distance of short-tailed shearwaters (*Puffinus tenuirostris*) was shorter during the rearing period [68]. However, few studies have targeted the diet of shearwaters during the prebreeding period. This study detected detailed diet information during the prebreeding period and observed differences between the prebreeding period and the rearing period. In the present study, the number of taxa detected per fecal sample during the rearing period was significantly higher than that during the prebreeding period (Fig 3B). The results of the NMDS analysis also indicated dietary dissimilarities between the prebreeding and rearing periods (Fig 4). Furthermore, all fecal samples were roughly divided into two main clusters, and fecal samples collected during the rearing period were lightly dispersed in the cluster analysis (Fig 3A). We deduced three plausible reasons for the cluster separation and the dispersion of rearing period samples: (a) seasonal fluctuations in the available prey resources, (b) variations in the foraging range of the wedge-tailed shearwater, and (c) a seasonal change in prey selection. The plausible reasons for the differences in the cluster dispersion between the prebreeding period and rearing period are described in the following paragraphs.

Although more information about the distribution and biomass of fishes and mollusks around the Ogasawara Islands is required, we can speculate that there are relationships between shearwater diet composition and the seasonal availability of prey. Exocoetidae genera, such as *Exocoetus* and *Cheilopogon*, were among the dominant fish prey taxa, which were also detected in some morphological studies [34,72]. *Exocoetus* was mainly detected during the prebreeding period, and *Cheilopogon* was detected during the rearing period. The Exocoetidae genera that were detected in the present study inhabit seas adjacent to the Ogasawara Islands [73]; however, the ecology of each Exocoetidae species is not well known. Seasonal differences among the detected Exocoetidae genera may reflect different patterns of migration and/or spawning. Large fishes were also detected, such as *Ranzania* during the prebreeding period and *Thunnus* during the rearing period. It appears that shearwaters may have fed upon the spawn, larvae, or carcasses of these large fishes, and their spawning periods may, therefore, have affected shearwater diet composition. The composition of staple mollusk prey also clearly differed between the prebreeding and rearing periods. According to Ando et al. (2004), *Ommastrephes bartrami* in the North Pacific Ocean is separated into spring and autumn populations [74]. The spring population spawns and hatches from winter to spring and migrates to the seas surrounding the Ogasawara Islands from January to June. The autumn population spawns and hatches from summer to autumn and migrates to the Ogasawara Islands from October to December [74]. Wedge-tailed shearwaters could have consumed mainly the spring population of *Ommastrephes*, and samples detected in feces collected during the rearing period may have belonged to early-arriving members of the autumn population. In a previous fishery report, *Sthenoteuthis oualaniensis* was caught from September to December [74], and this species is assumed to be an important resource for seabirds in the Ogasawara area. Indeed, *Sthenoteuthis* sequences dominated during the rearing period.

Changes in foraging range may be another important factor that contributes to the seasonal shift in diet composition. Streaked shearwaters (*Calonectris leucomelas*), which are widely distributed around Japanese islands, change their foraging areas in response to seasonal changes in the marine environment [70]. There are also some studies targeting wedge-tailed shearwaters at other breeding sites [60,75–77]. It is known that wedge-tailed shearwaters change foraging areas and strategies depending on the purpose of foraging, whether it is provision for themselves or their chicks [60,76]. In the case of this study, cluster analysis suggested that prey items detected from fecal samples from the rearing period were more diverse than those from the prebreeding period (Fig 3A and 3B). This result may indicate that wedge-tailed shearwaters needed to forage for a variety of prey items or expand their foraging area, perhaps because prey biomass decreased during the rearing period [78] or the nutrient requirements of chicks changed [79]. It is known that wedge-tailed shearwaters perform dual foraging excursion, which is a combination of long and short trips [75,77]. Increases in prey diversity during the rearing period may be caused by dual foraging excursions.

Variation in prey selection may also affect the seasonal differences in prey composition. Plasticity of prey selection in relation to prey availability and provision for themselves or their chicks has been identified in some species of seabirds [80,81], and this plasticity is possible for wedge-tailed shearwaters. Differences in major prey items between the two seasons may also reflect changes in shearwater foraging range and/or prey selection. Further analysis of available food resources and tracking foraging behavior may improve our understanding of wedge-tailed shearwater foraging strategies.

## Conclusion

In the present study, we estimated the composition of the wedge-tailed shearwater diet using a DNA metabarcoding approach. Based on this method, we filled gaps in the current knowledge of the shearwater diet during the prebreeding period. Our results indicate the importance of deep-sea fishes as stable food resources for shearwaters throughout the sampling seasons, although diet composition significantly differed between the prebreeding and rearing periods. Further investigations by tracking individual foraging behavior may improve our knowledge of wedge-tailed shearwater diet composition as it relates to foraging strategies.

The DNA metabarcoding approach is sensitive, and its application to fecal samples does not disturb seabirds because it is noninvasive, which facilitates diet monitoring. Long-term and wide-ranging studies of seabird diets using DNA metabarcoding may be useful for detecting changes in marine ecosystems and/or investigating the potential impacts of fisheries and climate changes.

## Supporting information

S1 Fig Rarefaction curves for each region and breeding phase. The effect of the sequencing effort on the estimated number of operational taxonomic units (OTUs). Each curve indicates a fecal sample, and numbers near the curves indicate the number of overlapping samples. (a) The 12S region targeting fishes during the prebreeding period, (b) the 12S region targeting fishes during the rearing period, (c) the 16S region targeting mollusks during the prebreeding period, and (d) the 16S region targeting mollusks during the rearing period.
(EPS)

S2 Fig Sample-based rarefaction curves for each breeding phase. The effect of fecal sampling effort on the estimated number of OTUs in the 12S and 16S regions. The shaded areas represent confidence intervals, and the curves within the shaded areas represent the average number of OTUs determined by random sampling. (a) Prebreeding period; (b) rearing period.
(EPS)
